# Supplementary material for: Seminoma and Embryonal Carcinoma Footprints Identified by Analysis of Integrated Genome-Wide Epigenetic and Expression Profiles of Germ Cell Cancer Cell Lines
Source: PLoS One. 2014 Jun 2;9(6):e98330. doi: 10.1371/journal.pone.0098330 (PMC4041891; doi:10.1371/journal.pone.0098330)
Supplement: File S1 — ZIP file containing DMRforPairs output for significant regions. Please start from the html files. (ZIP) [file pone.0098330.s008.zip › figures/9371.pdf]

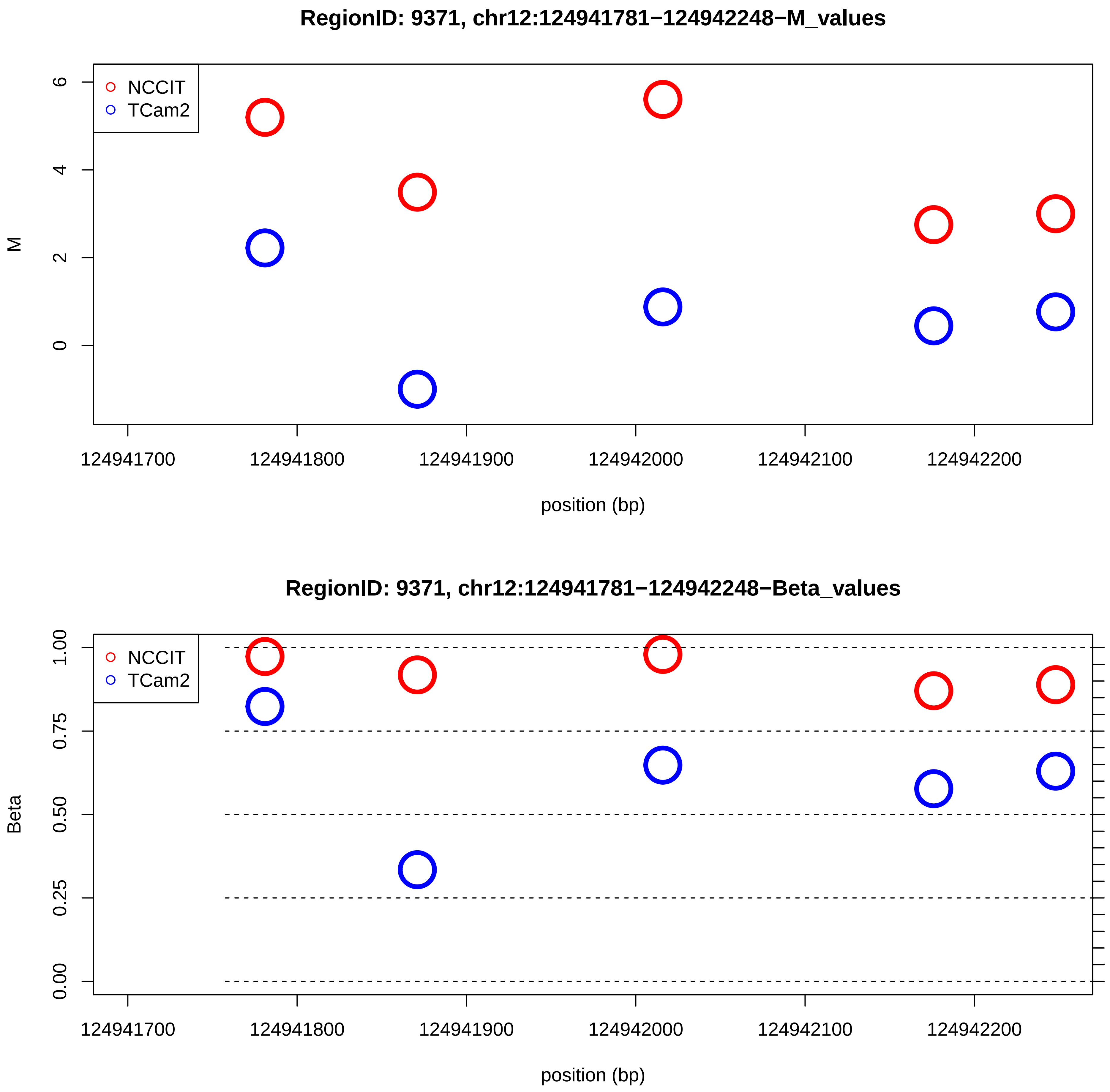
RegionID: 9371, chr12:124941781−124942248−M_values
NCCIT
TCam2
M
6
4
2
0
124941700
124941800
124941900
124942000
124942100
124942200
position (bp)
RegionID: 9371, chr12:124941781−124942248−Beta_values
NCCIT
TCam2
Beta
1.00
0.75
0.50
0.25
0.00
124941700
124941800
124941900
124942000
124942100
124942200
position (bp)
